# Curcumin Ameliorated Oxidative Stress and Inflammation-Related Muscle Disorders in C2C12 Myoblast Cells

**DOI:** 10.3390/antiox10030476

**Published:** 2021-03-17

**Authors:** Da-Yeon Lee, Yoon-Seok Chun, Jong-Kyu Kim, Jeong-Ok Lee, Young-Joon Lee, Sae-Kwang Ku, Soon-Mi Shim

**Affiliations:** 1 Department of Food Science and Biotechnology, Sejong University, 209, Neungdong-ro, Gwangjin-gu, Seoul 05006, Korea; dayeons93@naver.com; 2 Aribio H&B Co., Ltd., #710, Yongin Techno Valley, 357, Guseong-ro, Giheung-gu, Yongin-si, Gyeonggi-do 16914, Korea; yschun@aribiohnb.com (Y.-S.C.); jkkim@aribiohnb.com (J.-K.K.); ceo@aribiohnb.com (J.-O.L.); 3 Department of Preventive Medicine, Daegu Haany University, 1, Hanuidae-ro, Gyeongsan-si, Gyeongsangbuk-do 38610, Korea; gksxntk@dhu.ac.kr; 4 Department of Anatomy and Histology, Daegu Haany University, 1, Hanuidae-ro, Gyeongsan-si, Gyeongsangbuk-do 38610, Korea; gucci200@hanmail.net

**Keywords:** spray dry powder containing 40% curcumin (CM-SD), antioxidant, anti-inflammatory, oxidative stress, Nrf2, C2C12 myoblast cells

## Abstract

The purpose of the current study was to investigate antioxidant and anti-inflammatory effects of spray dry powder containing 40% curcumin (CM-SD) in C2C12 myoblast cells. CM-SD increased DPPH radical scavenging activity in a dose-dependent manner, and up to 30 μg/mL of CM-SD did not express cytotoxicity in C2C12 cells. Exposure to hydrogen peroxide (H_2_O_2_) drastically decreased the viability of C2C12 cells, but pre-treatment of CM-SD significantly increased the cell viability (*p* < 0.01). CM-SD significantly transactivated the nuclear factor erythroid-2-related factor 2 (Nrf2)-dependent luciferase activity in a dose-dependent manner and enhanced the levels of heme oxygenase (HO)-1, glutamate cysteine ligase catalytic subunit (GCLC), and NAD(P)H-dependent quinone oxidoreductase (NQO)-1. CM-SD also significantly reduced reactive oxygen species (ROS) production and lipid peroxidation and restored glutathione (GSH) depletion in H_2_O_2_-treated C2C12 cells. Moreover, CM-SD significantly reduced lipopolysaccharides (LPS)-mediated interleukin (IL)-6 production in the conditioned medium. Results from the current study suggest that CM-SD could be a useful candidate against oxidative stress and inflammation-related muscle disorders.

## 1. Introduction

Sarcopenia is the degenerative loss of skeletal muscle mass (0.5–1% loss per year after the age of 50), quality, and strength associated with aging [1]. Nutritional imbalance, accumulation of aged proteins, depletion of growth hormones, proinflammatory cytokines, oxidative stress-mediated apoptosis of skeletal muscles, and inactivation of satellite cells have been known to cause sarcopenia [2,3]. Although the detailed molecular mechanisms which contribute to sarcopenia are largely unknown and need to be further elucidated, recent evidence indicates that apoptosis is associated with the aging-related loss of skeletal muscle since increases in Bax/Bcl-2 ratio, cytoplasmic release of cytochrome c, activation of p53 and caspases, and fragmentation of chromosomal DNA are observed in aged muscles [4,5,6]. The current estimates suggest that about 200 million people worldwide will experience sarcopenia to a degree that could affect their health over the next four decades [7]. Although there are no approved mediations for the treatment of sarcopenia, exercise and nutrition for stimulating muscle protein synthesis have been suggested to delay the progression of sarcopenia. Thus, the development of strategies to counteract the negative impact of sarcopenia is warranted. Medicinal herbs are gaining space and importance in the pharmaceutical industry as well as inspiring the search for new potential sources of bioactive molecules, and they are considered to be potential sources to combat various diseases including muscle weakness [8,9,10,11,12]. As regulation of oxidative stress seems to be essential for sarcopenia [3,9], traditional herbs possessing potent antioxidant activities are attractive resources for managing sarcopenia with fewer side effects.

*Curcuma longa* L., turmeric or curcuma, an herbaceous perennial of the *Zingiberaceae*, is distributed in Africa, Latin America, and Asia, including India, China, and Japan [13,14]. Curcumin, a representative substance from the root of turmeric, includes about 2–8% turmeric and is consumed daily throughout Asian countries reported without toxicity [15,16]. Curcumin has also been reported to possess diverse pharmacological effects including antioxidant and anti-inflammatory activities [15,17]. Recently, it has been suggested that curcumin alleviated chronic kidney disease-induced muscle atrophy by inhibiting glycogen synthase kinase (GSK)-3β [18]. Nevertheless, the effects of curcumin on wasting muscle have not been fully established yet. Therefore, the objective of the present study was to evaluate in vitro antioxidant and anti-inflammatory effects of curcumin in a murine myoblast C2C12 cell lines, as a process to develop potent myoprotective medicinal foods.

## 2. Materials and Methods

### 2.1. Preparation of Test Articles

Spray dry powders of turmeric containing 40% curcumin (CM-SD) were supplied by Aribio Co., Ltd. (Seongnam-si, Gyeonggi-do, Republic of Korea) on 17 October 2018. In brief, commercial ethanol extract of turmeric (Curcumin C3 Complex^®^, 95% curcuminoids; Sabinsa Korea Corporation, Seoul, Republic of Korea) was blended with purified water, phosphoric acid, and Ghatti gum, and then the mixture was suspended for more than 20 h for particle sizes less than 1000 nm. After filtering, 6.76% of dextrin was added to the suspension and spray dried to produce Ari C4 Complex as CM-SD with 400 mg/g of curcumin. Some specimens of turmeric were deposited in the herbarium of the Department of Anatomy and Histology, College of Korean Medicine, Daegu Haany University. One hundred milligrams of CM-SD were dissolved in 1 mL of dimethyl sulfoxide (DMSO) and then filter-sterilized by using a 0.2 μm syringe filter (Nalgene, NY, USA). One hundred millimolar of 6-hydroxy-2,5,7,8-tetramethylchroman-2-carboxylic acid (Trolox; Merck Millipore, Burlington, MA, USA) and 30 μM of isorhamnetin (IsoR), a reference drug as a positive control, were also dissolved in DMSO. All test articles were stored at –20 °C to protect light and humidity until use.

**Figure 1:**
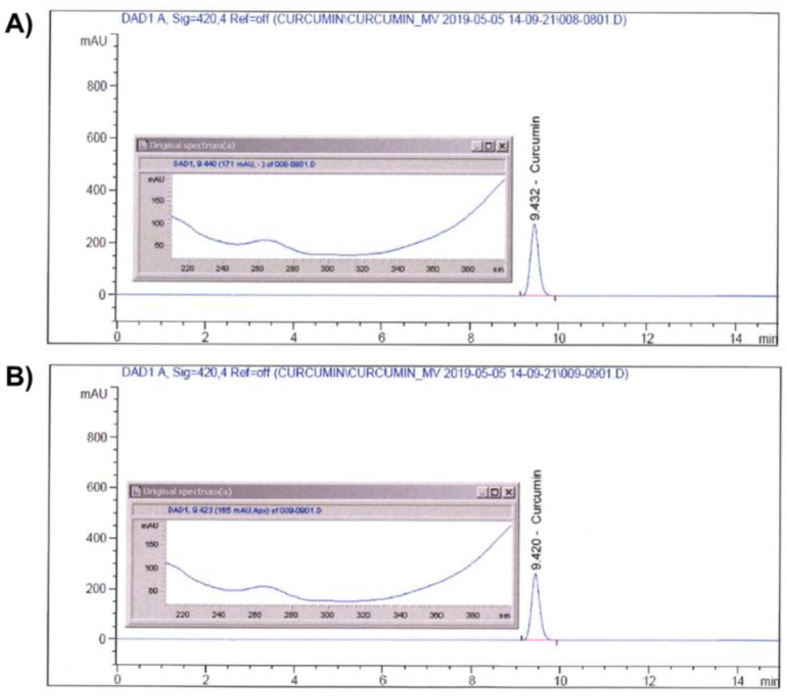
Identification of curcumin on standard (**A**) and CM-SD (**B**) by HPLC.

Identification of curcumin in CM-SD was performed by an Agilent 1100 Series high-performance liquid chromatography (HPLC) System (Agilent Technologies, Inc., Santa Clara, CA, USA) coupled with diode array detector. Curcumin was analyzed by a Capcell Pak^®^ C18 column (4.6 × 250 mm, 5 μm; Shiseido Co., Ltd., Kyoto, Japan) at 40 °C. A mobile phase containing 0.1% (*v*/*v*) phosphoric acid (Samchun Chemicals Co., Ltd., Pyeongtaek-si, Gyeonggi-do, Republic of Korea) (A) and acetonitrile (HPLC grade; Burdick & Jackson, Charlotte, NC, USA) (B) was used with the following gradient: 0–15 min, 50% B. The sample injection volume was 10 μL with a flow rate of 1 mL/min. The UV wavelength was set at 420 nm. Curcumin standard (Sigma-Aldrich, St. Louis, MO, USA) and CM-SD were both dissolved in HPLC grade methanol (Daejung Chemicals & Materials, Siheung-si, Gyeonggi-do, Republic of Korea) for the analyses. The chromatograms of curcumin in standard chemical and CM-SD are shown in Figure 1A,B, respectively.

### 2.2. Determination of Radical Scavenging Activity

Radical scavenging activity was determined using the 2,2-diphenyl-1-picrylhydrazyl (DPPH) method [19]. DPPH (Sigma-Aldrich) in ethanol solution (150 μM) was reacted with CM-SD (1–100 μg/mL) or Trolox (100 μM), a water-soluble analog of vitamin E. Samples were incubated at room temperature for 30 min under light protection. The absorbance was measured at 517 nm using a Synergy HTX microplate reader (BioTek Instruments, Inc., Winooski, VT, USA). Radical scavenging activity was calculated according to the following equation:

(1) $$\mathrm{Radical}\;\mathrm{scavenging}\;\mathrm{activity}\;\left(\%\right)\;=\;\left[1-\frac{\left(S-S_{0}\right)}{\left(C-C_{0}\right)}\right]\times 100,$$

where S and S_0_ indicate absorbance of DPPH solution with or without samples, and C and C_0_ indicate absorbance of solvent with or without sample.

### 2.3. C2C12 Myoblast Cell Culture

C2C12 cells, a murine myoblast cell line, were obtained from American Type Culture Collection (ATCC, Manassas, VA, USA) and maintained under standard conditions, as previously described [20]. Briefly, C2C12 myoblast cells were cultured in growth medium comprising 90% Dulbecco’s modified Eagle’s medium, 10% fetal bovine serum, 50 units/mL penicillin, and 50 μg/mL streptomycin at 37 °C in a humidified atmosphere with 5% CO_2_. When C2C12 cells were grown to about 70% of confluency, the cells were sub-cultured on appropriated new culture dishes to maintain phenotype of the myoblast cells. Recombinant HepG2 cells expressing antioxidant response element (ARE)-driven luciferase were generated, as described previously [21]. Briefly, pGL4.37[luc2P/ARE/Hygro] Vector, ARE-driven reporter gene construct, was obtained from Promega Corporation (Madison, WI, USA). HepG2 cells were stably transfected with pGL4.37 plasmid using FuGENE^®^ HD Transfection Reagent (Promega) according to manufacturer’s instruction, and 80 μg/mL of hygromycin was added to select the resistant colonies. The selected resistant colonies were pooled and used for reporter gene analysis.

### 2.4. Cell Viability Assay

C2C12 cells were seeded at a density of 2 × 10^4^ cells/well in a 96-well plate to determine the cytotoxicity activity of CM-SD. To examine the cytotoxicity of CM-SD, C2C12 cells were treated with 1–30 μg/mL of CM-SD for 24 h. To investigate the cytoprotective effects of CM-SD in C2C12 cells, 1–30 μg/mL of CM-SD were pre-incubated 1 h prior to the addition of 100 μM of hydrogen peroxide (H_2_O_2_; Sigma-Aldrich) for 6 h. Thirty micromolar of IsoR was used as a reference drug [22]. After incubation of the cells, viable cells were stained with 3-(4,5-dimethylthiazol-2-yl)-2,5-diphenyltetrazolium bromide (MTT, 0.1 μg/mL; Sigma-Aldrich) for 2 h, according to a previous study with some modifications [23]. The media were removed, and then the produced formazan crystals in each well were dissolved by 200 μL of DMSO. Absorbance was measured at 570 nm using an automatic microplate reader (BioTek Instruments, Inc.). Relative cell viabilities were calculated according to the following equation:

(2) $$\mathrm{Relative}\;\mathrm{cell}\;\mathrm{viability}\;\left(\%\;\mathrm{of}\;\mathrm{control}\right)\;=\;\frac{\mathrm{Absorbance}\;\mathrm{of}\;\mathrm{treated}\;\mathrm{sample}}{\mathrm{Absorbance}\;\mathrm{of}\;\mathrm{control}}\times 100,$$

### 2.5. Nuclear Factor Erythroid-2-Related Factor 2 (Nrf2)-Dependent Reporter Gene Assay

To determine the luciferase activity, recombinant HepG2 cells which express pGL4.37 were replated in 12-well plates (5 × 10^5^ cells/well) overnight and exposed to 1–30 μg/mL of CM-SD for 18 h, as previously described [21]. The relative luciferase activity was calculated as the relative change to protein content measured by the bicinchoninic acid method. To investigate Nrf2 transactivation in C2C12 cells, reporter gene assay was carried out as previously established [24]. Briefly, C2C12 cells were plated at a density of 5 × 10^4^ cells per well in a 12-well plate and allowed to grow overnight. The cells were co-transfected with 500 ng of pGL4.37 and 100 ng of pRL-TK (*Renilla* luciferase expression plasmid under an HSV-thymidine kinase promoter) for 6 h in the presence of FuGENE^®^ HD transfection reagent (Promega), and then exposed to 3–30 μg/mL of CM-SD for 18 h. Thirty micromolar of IsoR was used as a reference drug [22]. The luminescence intensity of treated cell lysates was measured using Dual-Luciferase Reporter Assay System and GloMax^®^ 20/20 Luminometer (Promega). The luminescence intensity of firefly luciferase was divided by that of *Renilla* luciferase to calculate the relative luciferase activity.

### 2.6. Total RNA Isolation and Real-Time Reverse Transcription Polymerase Chain Reaction (RT-PCR)

After C2C12 cells were treated with either 3–30 μg/mL of CM-SD or 30 μM of IsoR for 12 h, total RNA was isolated from treated cells using Tri-solution (Bioscience Technology, Daegu, Republic of Korea). To obtain cDNA, the RNA (2 μg each) and oligo-d(T)_16_ primer were added to AccuPower^®^ RT PreMix (Bioneer Corporation, Daejeon, Korea), and resulting mixtures were reverse-transcribed using a Mastercycler^®^ Nexus Gradient Thermal Cycler (Eppendorf, Hamburg, Germany). Real-time PCR was performed using a CFX-96 (Bio-Rad, Hercules, CA, USA) and a SYBR^®^ Green Premix (Takara Bio Inc., Shiga, Japan). Glyceraldehyde-3-phosphate dehydrogenase (GAPDH) was used as a reference gene and melting curve analysis was performed to verify amplicon accuracy. Relative expression of each gene was calculated as 2^−ΔΔCT^, as previously reported [25]. Primers for the amplification of specific genes were synthesized from Bioneer Corporation, and nucleotide sequences are listed in Table 1.

### 2.7. Determination of Superoxide Dismutase (SOD) Activity

After C2C12 cells were treated with either 3–30 μg/mL of CM-SD or 30 μM of IsoR for 24 h, cells in phosphate buffered saline were homogenized by sonication and clarified by centrifugation at 1500× *g* for 10 min. SOD activity in cell homogenates was determined using a commercial kit (Cayman Chemical, Ann Arbor, MI, USA) which utilizes a tetrazolium salt for detection of superoxide radicals generated by xanthine oxidase and hypoxanthine at 450 nm. SOD activity was determined from a standard curve, and specific activity was obtained by dividing SOD activity by protein concentration.

### 2.8. Determination of Glutathione (GSH) Contents

Glutathione contents were determined using GSH/GSSG-Glo™ Assay Kit (Promega), according to manufacturer’s instructions. Briefly, C2C12 cells were pretreated with either 3–30 μg/mL of CM-SD or 30 μM of IsoR for 1 h, and then exposed to 100 μM of H_2_O_2_ for 6 h. Cells were lysed with glutathione lysis reagent and then incubated with luciferin generating reagent for 30 min and luciferin detection reagent for 15 min. Luminescence intensities were measured using an automated microplate reader (Infinite 200 PRO; Tecan Group Ltd., Männedorf, Switzerland), and the concentration of GSH was determined from a standard curve.

### 2.9. Determination of Reactive Oxygen Species (ROS) Production

ROS production was analyzed as previously described [26] with some modifications. Briefly, C2C12 cells were replated in 96-well black plate (2 × 10^4^ cells/well) overnight, pretreated with either 3–30 μg/mL of CM-SD or 30 μM of IsoR for 1 h, and exposed to 100 μM of H_2_O_2_ and 1 μM of 2′,7′-dichlorofluorescein diacetate (DCFH-DA; Sigma-Aldrich) for 3 h. Thirty micromolar of IsoR was used as reference drug. The fluorescence emitted by dichlorofluorescein was measured at excitation/emission wavelengths of 485/530 nm using an automated microplate reader (Tecan Group Ltd.).

### 2.10. Determination of Malondialdehyde (MDA) Contents

C2C12 cells were pretreated with either 3–30 μg/mL of CM-SD or 30 μM of IsoR for 1 h, and subsequently exposed to 100 μM of H_2_O_2_ for 12 h. After treatment, the cells were lysed by a radioimmunoprecipitation assay buffer containing 1 mM sodium fluoride, 1 mM β-glycerophosphate, 1 mM sodium orthovanadate, 2.5 mM sodium pyrophosphate, and protease inhibitor cocktail (GenDEPOT, Barker, TX, USA) for 1 h on ice and centrifuged at 15,000× *g* for 10 min. MDA levels in the resulting lysates (approximately 250 μg of protein lysates) were assayed according to the manufacturer’s instruction (TBARS Assay Kit; Cayman Chemical, Ann Arbor, MI, USA). The absorbance of the sample was monitored at 530 nm using a microplate reader (BioTek Instruments, Inc.), and the concentration of MDA was determined from a standard curve.

### 2.11. Quantification of Tumor Necrosis Factor (TNF)-α, Interleukin (IL)-1β, and Interleukin (IL)-6 Levels

To quantify proinflammatory cytokines in the conditioned medium, C2C12 cells were pretreated with 3–30 μg/mL of CM-SD or 30 μM of IsoR for 1 h and subsequently exposed to 1 μg/mL of lipopolysaccharide (LPS) for 12 h, as previously described [23]. Levels of murine TNF-α, IL-1β, and IL-6 in conditioned media were measured by BD OptEIA™ ELISA sets (BD Biosciences, San Diego, CA, USA), according to manufacturer instructions. Briefly, Nunc MaxiSorp™ 96-well microplate (Thermo Fisher Scientific, Waltham, MA, USA) was coated with each diluted capture antibodies, blocked with phosphate buffered saline containing 10% fetal bovine serum, bound with diluted conditioned media, and incubated with streptavidin-conjugated horseradish peroxidase and detection antibodies. After intense washing with phosphate buffered saline containing 0.05% Tween 20, the wells were developed with TMB Substrate Reagent Set (BD Biosciences) and added 2 N of H_2_SO_4_ to stop color development. Absorbance was measured at 420 nm with an automated microplate reader (BioTek Instruments). Absorbance at 570 nm was used as reference absorbance. Levels of IL-6 were calculated by interpolation of standard curve.

### 2.12. Statistical Analyses

All numerical data were expressed as mean ± standard deviation (SD) from at least three separated experiments. One-way analysis of variance test (ANOVA) was used to determine the significances of differences among experimental groups, and Levene’s test was used to examine variance homogeneity. Fisher’s least significant difference (LSD) test for variance homogeneity or Dunnett’s T3 test for variance non-homogeneity was used as post hoc analysis to identify significant differences between pairs of groups. Differences were considered significant at *p* < 0.05. Statistical analyses were conducted using SPSS for Windows (Release 14.0K; SPSS Inc., Chicago, IL, USA).

## 3. Results and Discussion

### 3.1. Radical Scavenging Activity of CM-SD

**Figure 2:**
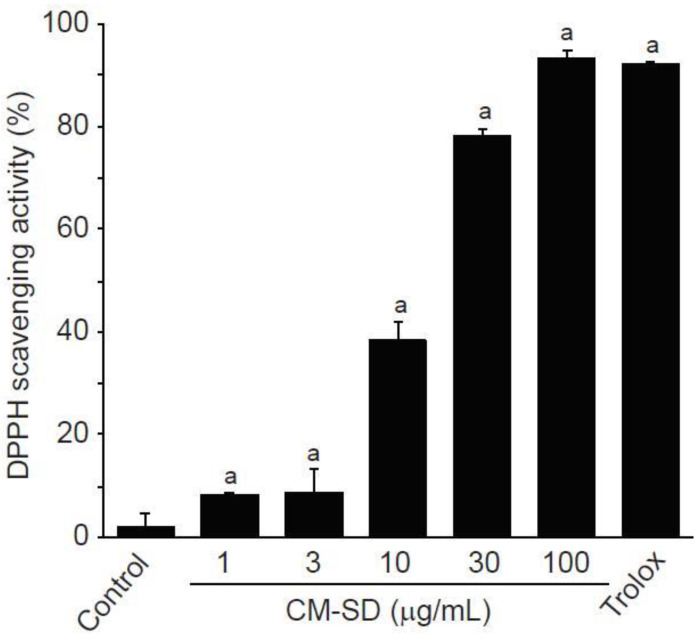
DPPH scavenging activity of curcumin 40% spray dry powders (CM-SD). Values are expressed as mean ± SD of three separate experiments. ^a^ *p* < 0.01, significant as compared with control by LSD test.

Antioxidant activity is regarded as one of the most important mechanisms for the regulation of degenerative loss of skeletal muscle. To investigate in vitro antioxidant capacity of CM-SD, the current study examined radical scavenging activity by DPPH assay. CM-SD increased radical scavenging activity in a dose-dependent manner, as shown in Figure 2. Relative radical scavenging activities by 1, 3, 10, 30, and 100 μg/mL of CM-SD were 8.19 ± 0.47%, 8.85 ± 4.49%, 35.25 ± 3.67%, 78.42 ± 1.02%, and 93.53 ± 1.38%, respectively. Radical scavenging activity by 100 μM of Trolox was 92.30 ± 0.17%. Significant differences in radical scavenging activity were observed in 1–100 μg/mL of CM-SD, and the level for 100 μg/mL of CM-SD was comparable to that by 100 μM of Trolox (*p* = 0.562 in LSD test).

Enhancing antioxidant capacity plays an essential role in protecting cells from oxidative stress and reducing inflammatory responses in skeletal muscle. Therefore, we firstly investigated the antioxidant potential of CM-SD using in vitro DPPH radical, a well-known radical generator. Results from the present study exhibited that CM-SD increased radical scavenging activity in a dose-dependent manner. Similarly, a previous study using in vitro models found that curcumin proved antioxidant and free radical scavenging abilities [27]. Thus, CM-SD seems to have potent radical scavenging activities without any cytotoxicity at least in our experimental system, while further studies would be required to evaluate in vitro and in vivo toxicity studies for CM-SD.

### 3.2. Effects of CM-SD on C2C12 Cell Viability and H_2_O_2_-Induced Cytotoxicity

**Figure 3:**
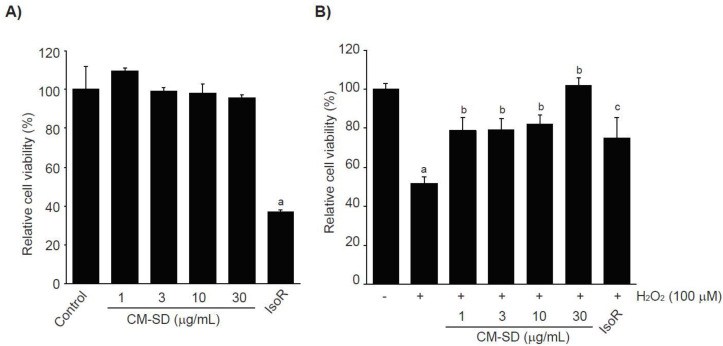
Effect of CM-SD on cell viability (**A**) and H_2_O_2_-mediated cytotoxicity (**B**) of C2C12 cells. Values are expressed as mean ± SD of six separate experiments. ^a^ *p* < 0.01, significant as compared with control by Dunnett’s T3 test; ^b^ *p* < 0.01, ^c^ *p* < 0.05, significant as compared with H_2_O_2_-treated cells by Dunnett’s T3 test.

Prior to evaluating the antioxidant potentials, the effects of CM-SD on cell viabilities were investigated after treating C2C12 cells with 1–30 μg/mL of CM-SD for 24 h. The results from MTT assay indicate that treatment with CM-SD did not show any cytotoxicity. However, treatment of IsoR (30 μM, 24 h), a reference drug, significantly decreased the viability of C2C12 cells (Figure 3A). Relative cell viability by treatment with 1, 3, 10, and 30 μg/mL of CM-SD was 109.60 ± 1.55%, 99.37 ± 1.89%, 98.38 ± 4.69%, and 95.74 ± 1.66% of control cells, respectively. Cell viability by 30 μM of IsoR was 37.19 ± 0.55% of control cells. Since 1–30 μg/mL of CM-SD did not show any cytotoxicity, the current study chose up to 30 μg/mL of CM-SD as a maximum treatment concentration in subsequent experiments in C2C12 cells.

It was well established that H_2_O_2_, lactate, and LPS are representative agents to provoke cytotoxicity of myoblasts by inducing oxidative stress, metabolic stress, and inflammation [28,29,30,31], The current study preliminary tested whether H_2_O_2_, sodium L-lactate (20 mM), or LPS (1 μg/mL) reduced the viability of C2C12 cells or not. Only treatment of H_2_O_2_ (1 mM, 24 h) significantly decreased the viability of C2C12 cells (data not shown). To examine the effect of CM-SD on oxidative stress-mediated cytotoxicity, C2C12 cells were pretreated with 1–30 μg/mL of CM-SD for 1 h, subsequently exposed to 100 μM of H_2_O_2_ for 6 h, and then determined the cell viability by MTT assay. Exposure of H_2_O_2_ for 6 h significantly decreased the cell viability (51.80 ± 3.36% of control). However, pretreatment of CM-SD increased cell viability, and the cell viability by pretreatment with 30 μg/mL of CM-SD was comparable to control cells (*p* = 0.998). Statistical significances of differences were observed in all four different concentrations of CM-SD-pretreated cells as compared with H_2_O_2_ alone-treated cells (Figure 3B). When comparing with 30 μM of IsoR, pretreatment of CM-SD (30 μg/mL) showed a more potent cytoprotective activity against H_2_O_2_-induced cytotoxicity (*p* = 0.013). Relative cell viability by pretreatment with 1, 3, 10, and 30 μg/mL of CM-SD were 78.91 ± 6.47%, 79.39 ± 5.55%, 81.97 ± 4.97%, and 101.87 ± 3.78% of control cells, respectively. Relative cell viability by pretreatment with 30 μM of IsoR was 74.97 ± 10.67% of control cells.

Hydrogen peroxide (H_2_O_2_), generated in oxidative stress, easily penetrates inside and outside of the cells and acts as a second messenger for maintaining redox homeostasis [32]. However, H_2_O_2_, beyond adaptation capacity, produces more aggressive oxygen radicals, leads to dysfunction of biological macromolecules, and thereby induces DNA damage, apoptosis, and necrosis [33]. In this regard, H_2_O_2_ has been widely used as a pro-oxidant in in vitro cultured cell lines including C2C12 cells [22]. In parallel with the previous report, the present results showed that the treatment of 100 μM of hydrogen peroxide for 6 h significantly reduced the viability of C2C12 cells. However, pretreatment of CM-SD concentration-dependently inhibited the H_2_O_2_-mediated cytotoxicity.

### 3.3. Effects of CM-SD on Transcription of Antioxidant Genes

**Figure 4:**
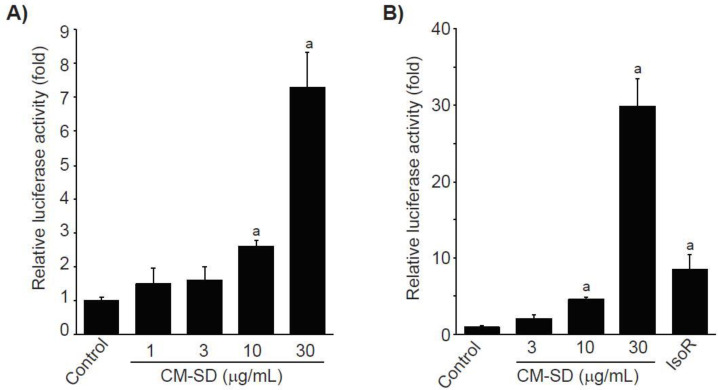
Effect of CM-SD on Nrf2 transactivation in recombinant HepG2 cells (which harbor ARE-driven reporter gene) (**A**) and C2C12 cells (**B**). Values are expressed as mean ± SD at least three separate experiments. Experimental data from HepG2 and C2C12 cells were analyzed by LSD and Dunnett’s T3 test, respectively. ^a^ *p* < 0.01, significant as compared with control.

To examine whether Nrf2 is involved in CM-SD-mediated cytoprotectant, recombinant HepG2 cells, which harbor the ARE-driven reporter gene, were treated with 1–30 μg/mL of CM-SD for 18 h, and then the CM-SD-mediated luciferase activities were monitored. Results from reporter gene assay indicate that treatment with CM-SD significantly increased ARE-driven luciferase activities in a concentration-dependent manner. Statistical significances of differences were observed in 10 and 30 μg/mL of CM-SD-treated cells as compared with control cells (Figure 4A). Relative luciferase activities by treatment with 1, 3, 10, and 30 μg/mL of CM-SD were 1.48 ± 0.46, 1.60 ± 0.39, 2.60 ± 0.17, and 7.30 ± 1.02 folds of control cells, respectively. To further confirm whether CM-SD transactivates Nrf2 in C2C12 cells, C2C12 cells were transiently co-transfected with pGL4.37 and pRL-TK reporter plasmids and treated with either 3–30 μg/mL of CM-SD or 30 μM of IsoR for 18 h. In parallel with the results obtained from recombinant HepG2 cells, treatment with 10 and 30 μg/mL of CM-SD significantly increased Nrf2-mediated luciferase activity. When comparing with 30 μM of IsoR (8.53 ± 1.87-fold the control cells), 30 μg/mL of CM-SD showed a more potent Nrf2 transactivation (*p* = 0.002 in Dunnett’s T3 test) (Figure 4B). Relative luciferase activities treated with 3, 10, and 30 μg/mL of CM-SD were 2.04 ± 0.44, 4.52 ± 0.34, and 29.88 ± 3.64 folds of control cells, respectively.

**Figure 5:**
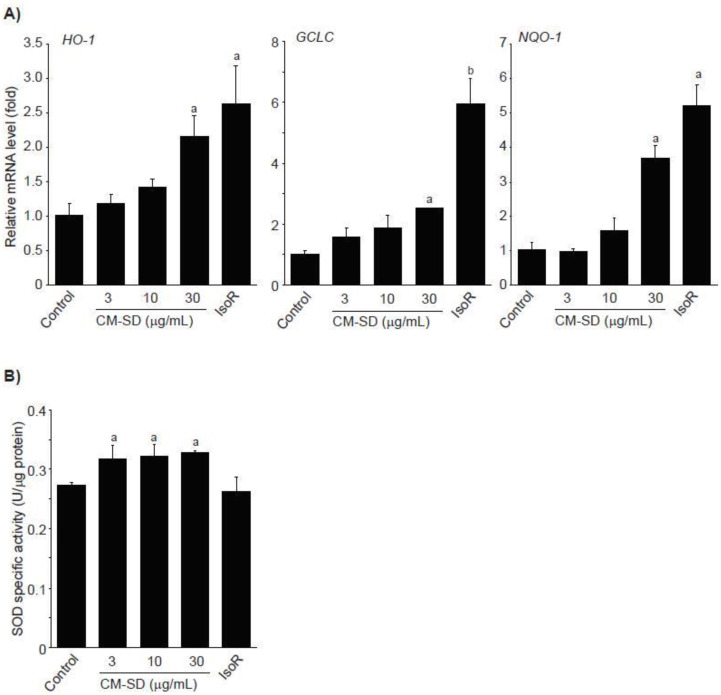
Effect of CM-SD on HO-1, GCLC, and NQO-1 activation (**A**) and SOD activity (**B**). Values are expressed mean ± SD of three separate experiments. Experimental data for HO-1, NQO-1, and SOD were analyzed by LSD test, and the data for GCLC were for Dunnett’s T3 test. ^a^ *p* < 0.01 and ^b^ *p* < 0.05, significant as compared with control.

Activated Nrf2 binds to ARE and enhances transcription of antioxidant genes such as heme oxygenase (HO)-1, glutamate cysteine ligase catalytic subunit (GCLC), NADH-dependent quinone oxidoreductase (NQO)-1, and SOD [34,35]. To examine whether Nrf2 activation by CM-SD increases activities of antioxidant genes, C2C12 cells were treated with 3–30 μg/mL of CM-SD for 12 h, and then monitored mRNA levels of antioxidant genes by real-time PCR analysis. Results from real-time PCR analysis indicate that treatment with CM-SD tended to increase mRNA levels of HO-1, GCLC, and NQO-1, while induction of antioxidant genes treated by 30 μg/mL of CM-SD was only statistically significant as compared with control cells. As expected, IsoR (30 μM) significantly increased the mRNA levels of HO-1, GCLC, and NQO-1 (Figure 5A). Relative HO-1 mRNA levels treated with 3 μg/mL CM-SD, 10 μg/mL CM-SD, 30 μg/mL CM-SD, and 30 μM IsoR were 1.19 ± 0.12, 1.41 ± 0.12, 2.16 ± 0.31, and 2.64 ± 0.56 folds of control cells, respectively. Relative GCLC mRNA levels treated with 3 μg/mL CM-SD, 10 μg/mL CM-SD, 30 μg/mL CM-SD, and 30 μM IsoR were 1.57 ± 0.31, 1.88 ± 0.38, 2.51 ± 0.02, and 5.94 ± 0.88 folds of control cells, respectively. Relative NQO-1 mRNA levels treated with 3 μg/mL CM-SD, 10 μg/mL CM-SD, 30 μg/mL CM-SD, and 30 μM IsoR were 0.96 ± 0.09, 1.58 ± 0.36, 3.69 ± 0.38, and 5.22 ± 0.60 folds of control cells, respectively. When C2C12 cells were treated with either 3–30 μg/mL of CM-SD or 30 μM of IsoR for 24 h, SOD activities in cell homogenates were marginally, but significantly increased by three different dosages of CM-SD. However, IsoR had no effect on SOD activity (Figure 5B). Specific activities of SOD in control, 3 μg/mL CM-SD-, 10 μg/mL CM-SD-, 30 μg/mL CM-SD-, and 30 μM IsoR-treated cells were 0.27 ± 0.005, 0.32 ± 0.02, 0.32 ± 0.02, 0.33 ± 0.003, and 0.26 ± 0.24 U/μg of protein, respectively.

Nrf2 is an essential master regulator that protects cells against oxidative stress and enhances the cellular defense system through the induction of antioxidant genes [36,37]. Activated Nrf2 is released from its cytosolic repressor Keap1, translocates into the nucleus, binds to ARE in the promoter regions, and then induces the expression of antioxidant genes [38,39]. Therefore, Nrf2 binding to the ARE is crucial for enhancing the antioxidant capacity of cells. In addition, it has been reported that Nrf2 knockout reduces cannabinoid type 2 receptor-mediated myoblast differentiation, which suggests Nrf2 contributes to the differentiation of myoblasts to myotubes [40]. Results from the ARE-driven reporter gene assay using recombinant HepG2 cells and C2C12 cells suggest treatment with CM-SD significantly increased ARE-driven luciferase activities in a concentration-dependent manner. As curcumin and polyphenolic compounds are well-known Nrf2 inducers [22,41], these compounds existing in CM-SD could contribute to activate Nrf2 and to protect cells from excess oxidative stress. Further studies are needed to elucidate essential compounds in CM-SD to activate Nrf2. In addition, it has been reported that a variety of cellular signaling pathways such as p38 mitogen-activated protein kinase, extracellular-regulated protein kinase, phosphatidylinositol 3-kinase, protein kinase C, or casein kinase are involved in activation of Nrf2 [34,42,43,44]. Therefore, upstream signaling molecules contributed to Nrf2 activation by CM-SD should also be elucidated in the future.

Expression of Nrf2-mediated target gene promotes cell survival in oxidizing environments via enhancement of free radical metabolism, regulation of proteasome function, maintaining of glutathione homeostasis, inhibition of cytokine-mediated inflammation, and recognition of damaged DNA [34]. It has been well-known that several antioxidant genes such as HO-1, GCLC, and NQO-1 contain AREs in their promoter regions, and they are induced by Nrf2 activation [34,36]. HO-1 is an inducible rate-limiting enzyme initially identified as facilitating degradation of heme to biliverdin, ferrous iron, and carbon monoxide. Because the final products (e.g., carbon monoxide) of heme degradation exert antioxidant effects by neutralizing oxidative stress, HO-1 induction represents a potential therapeutic target in the management of oxidative stress-related disorders [45]. GCLC, the first rate-limiting enzyme of glutathione biosynthesis, is also involved in the protection of cells from oxidative stress by accelerating GSH biosynthesis [46]. NQO-1 is a member of NAD(P)H dehydrogenase family and involves two-electron reduction of quinones to hydroquinones. Therefore, NQO-1 prevents the one electron reduction of quinonoid compounds that results in the production of reactive oxygen species [47]. In the present study, treatment with CM-SD tended to increase mRNA levels of HO-1, GCLC, and NQO-1. Another antioxidant enzyme such as SOD is also essential in both scavenging ROS and maintaining cellular integrity [48]. SOD diminishes ROS by conversion of superoxide radical to H_2_O_2_. The present results indicate that CM-SD marginally, but significantly, increased SOD activity. Taken together, CM-SD seems to enhance antioxidant capacity via activating Nrf2-dependent antioxidant gene induction.

### 3.4. Effects of CM-SD on H_2_O_2_-Mediated Oxidative Stress

**Figure 6:**
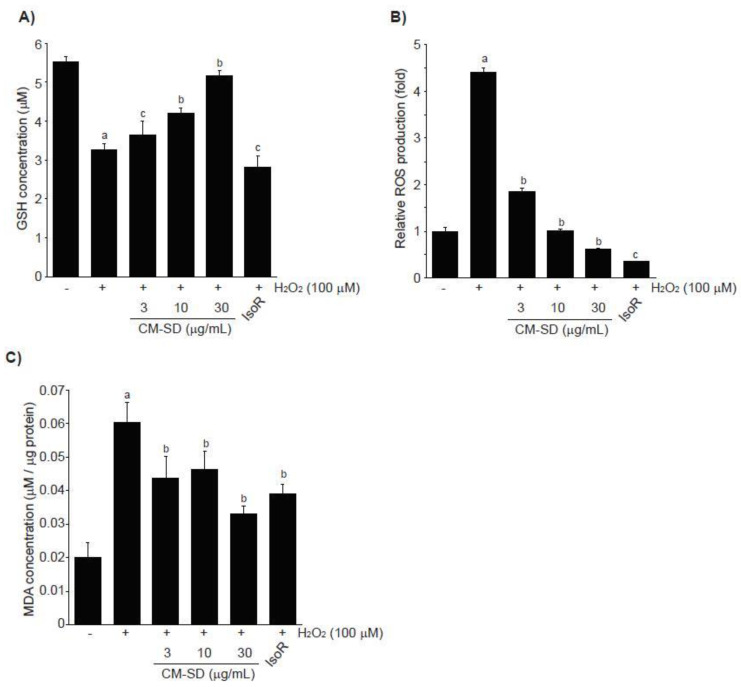
Effect of CM-SD on GSH (**A**), ROS (**B**), and MDA (**C**) levels followed by H_2_O_2_-induced oxidative stress in C2C12 cells. Values are expressed mean ± SD of three separate experiments. Experimental data for GSH and MDA were analyzed by LSD test, and the data for ROS were for Dunnett’s T3 test. ^a^ *p* < 0.01, significant as compared with control; ^b^ *p* < 0.01 and ^c^ *p* < 0.05, significant as compared with H_2_O_2_-treated cells.

Depletion of endogenous antioxidants (e.g., GSH) and acceleration of ROS production and lipid peroxidation are associated with H_2_O_2_-mediated cytotoxicity. To examine whether CM-SD-dependent activation of antioxidant defense system contributes to protect cells from H_2_O_2_-mediated oxidative stress, we first measured GSH levels in H_2_O_2_-exposed C2C12 cells. H_2_O_2_ (100 μM, 6 h) significantly depleted endogenous GSH in C2C12 cells. However, pretreatment with 3–30 μg/mL of CM-SD for 1 h concentration-dependently attenuated the depletion of intracellular GSH by H_2_O_2_. GSH levels with 30 μg/mL of CM-SD pretreatment were comparable to those of control cells (*p* = 0.077 in LSD test). IsoR (30 μM) did not alter the GSH reduction by H_2_O_2_ treatment (Figure 6A). GSH levels in control, H_2_O_2_, H_2_O_2_ + 3 μg/mL CM-SD-, H_2_O_2_ + 10 μg/mL CM-SD-, H_2_O_2_ + 30 μg/mL CM-SD-, and H_2_O_2_ + 30 μM IsoR-treated cells were 5.51 ± 0.14, 3.25 ± 0.19, 3.66 ± 0.33, 4.20 ± 0.12, 5.16 ± 0.14, and 2.81 ± 0.30 μM, respectively.

In addition, exposure of H_2_O_2_ for 3 h increased ROS production (4.42 ± 0.08 folds of control cells), and pretreatment with either 3–30 μg/mL of CM-SD or with 30 μM of IsoR significantly inhibited the ROS production by H_2_O_2_. Relative ROS levels in 3 μg/mL CM-SD-, 10 μg/mL CM-SD-, 30 μg/mL CM-SD-, and 30 μM IsoR-pretreated cells were 1.85 ± 0.07, 1.02 ± 0.02, 0.61 ± 0.02, and 0.37 ± 0.01 folds, respectively (Figure 6B). Moreover, pretreatment with 3–30 μg/mL of CM-SD significantly reduced MDA levels by H_2_O_2_ in a concentration-dependent manner, as compared with H_2_O_2_-treated cells (100 μM, 12 h). Inhibition of MDA with 30 μg/mL of CM-SD was comparable to that by 30 μM of IsoR (Figure 6C). MDA levels in control, H_2_O_2_, H_2_O_2_ + 3 μg/mL CM-SD-, H_2_O_2_ + 10 μg/mL CM-SD-, H_2_O_2_ + 30 μg/mL CM-SD-, and H_2_O_2_ + 30 μM IsoR-treated cells were 0.0200 ± 0.0047, 0.0603 ± 0.0057, 0.0437 ± 0.0067, 0.0463 ± 0.0056, 0.0331 ± 0.0024, and 0.039 ± 0.0031 μM/μg protein, respectively.

### 3.5. Effects of CM-SD on LPS-Induced Proinflammatory Cytokines Production

**Figure 7:**
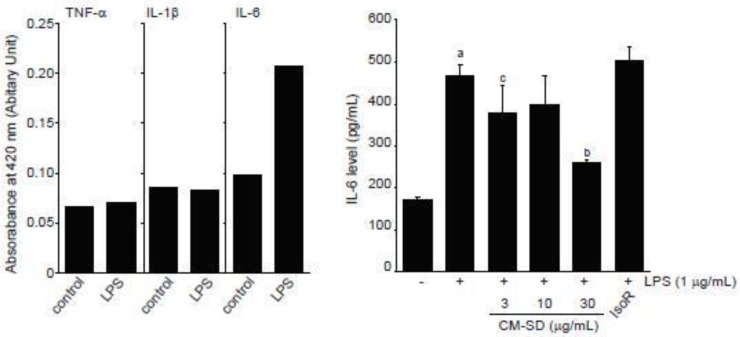
Effect of CM-SD on proinflammatory cytokines production. Values are expressed mean ± SD of three separate experiments. ^a^ *p* < 0.01, significant as compared with control by Dunnett’s T3 test; ^b^ *p* < 0.01, ^c^ *p* < 0.05, significant as compared with LPS-treated cells by Dunnett’s T3 test.

LPS is endotoxin of gram-negative bacteria that binds to toll-like receptor (TLR) 4 and activates an acute inflammatory response in myoblast as well as immune cells [31,49]. When C2C12 cells were exposed to 1 μg/mL of LPS for 12 h, our preliminary experiments by ELISA showed that IL-6, but not TNF-α and IL-1β, increased in conditioned medium obtained from LPS-treated C2C12 cells (Figure 7, left). To examine the effect of CM-SD on LPS-mediated proinflammatory cytokines production, C2C12 cells were pretreated with either 3–30 μg/mL of CM-SD or 30 μM of IsoR for 1 h, subsequently exposed to 1 μg/mL of LPS for 12 h, and then the IL-6 production in culture medium was determined. When compared to the control, treatment with LPS (1 μg/mL) significantly increased the production of IL-6. Pretreatment of CM-SD significantly decreased LPS-inducible IL-6 production. However, IsoR pretreatment did not inhibit IL-6 production by LPS (Figure 7, right). IL-6 levels in control, LPS alone, LPS + 3 μg/mL CM-SD-, LPS + 10 μg/mL CM-SD-, LPS + 30 μg/mL CM-SD-, and LPS + 30 μM IsoR-treated cells were 171.43 ± 6.23, 468.10 ± 27.86, 380.95 ± 62.14, 400.48 ± 64.81, 259.05 ± 7.87, and 502.38 ± 33.57 pg/mL, respectively.

Since myoblast cells have been known to constitutively express TLR4, an LPS receptor [31], LPS stimulates expression of proinflammatory cytokines in myoblast and myotube cells [50,51] and inhibits myogenic differentiation via activating TLR4/nuclear factor-kappa B (NF-κB) and secreting TNF-α [31]. Among numerous proinflammatory cytokines, IL-6 is a pleiotropic cytokine that is elevated by inflammatory response, exercise, muscle contraction, and denervation in skeletal muscle [50,52,53,54]. In the present study, we showed that CM-SD tended to decrease IL-6 secretion in the medium of LPS-stimulated C2C12 cells, and IL-6 reductions by 3 and 30 mg/mL of CM-SD treatment were statistically significant as compared to LPS alone treated cells. Therefore, these results raise the possibility that CM-SD has anti-inflammatory activity in myoblast cells. Unfortunately, our preliminary ELISA results could not detect secretion of TNF-α and IL-1β in the medium of LPS (1 μg/mL, 12 h)-stimulated C2C12 cells. Since TNF-α and IL-1β are known to be secreted directly from myoblasts after LPS stimulation [51], treatment condition limitations and detection methods may affect the differential results in TNF-α and IL-1β. Thus, more studies are still required to explore the effects of CM-SD on other proinflammatory cytokines in C2C12 cells.

Several studies assessed the inhibitory effect of curcumin against inflammation by modulating the macrophage response to LPS, which is a well-established, commonly used, and effective model for assessing inflammatory response in vitro. For instance, it was revealed that curcumin (1–10 μM) inhibited the expression of IL-1β and TNF-α in a dose-dependent manner in RAW 264.7 cells, further suggesting that curcumin could be a potential anti-inflammation agent [55]. Furthermore, LPS-induced TNF-α expression level was reduced by the pretreatment of 10 μM curcumin in RAW 264.7 cells [56]. Parallel with these findings, results from the current study confirm that pretreatment with 3–30 μg/mL of CM-SD potently inhibited expression of LPS-induced inflammatory cytokines, implying the prominent role of curcumin in the prevention of inflammation-related muscle illnesses.

Previously, it was reported that IsoR downregulates inflammatory responses via inhibition of c-Jun N-terminal protein kinase, Akt, and NF-κB in LPS-stimulated macrophages [57]. In addition, IsoR protects cells from oxidative stress by activating Nrf2-dependent antioxidant genes as well as AMP-activated protein kinase [58,59]. Moreover, IsoR induces extracellular regulated protein kinase-dependent Nrf2 activation and thereby scavenges H_2_O_2_-mediated oxidative stress in C2C12 cells [22]. Therefore, in the present study, IsoR (30 μM) was used as a reference drug to compare the beneficial effects of CM-SD in C2C12 cells. IsoR significantly reduced H_2_O_2_-mediated cytotoxicity, increased mRNA expression of Nrf2-dependent antioxidant genes, and decreased H_2_O_2_-mediated ROS and MDA productions in C2C12 cells. On the contrary, the present results indicate that IsoR alone showed severe cytotoxicity in C2C12 cells, while Choi [22] reported treatment with 30 μM of IsoR alone for 24 h did not change the cell viability. In addition, IsoR did not affect SOD activity, restore H_2_O_2_-mediated GSH depletion, or block LPS-inducible IL-6 production. Therefore, Nrf2-independent mechanisms as well as polymorphism of reference drug may differentially influence antioxidant and anti-inflammatory activities of IsoR in C2C12 cells. These issues should be further clarified for using IsoR as an antioxidant and anti-inflammatory nutraceutical in C2C12 cells.

## 4. Conclusions

The present study demonstrated that CM-SD in C2C12 myoblast cells showed in vitro antioxidant and anti-inflammatory activities. Specifically, CM-SD enhanced DPPH radical scavenging activity in a dose-dependent manner, and it did not affect the cell viability of C2C12 cells with concentrations up to 30 μg/mL. Furthermore, CM-SD significantly suppressed H_2_O_2_-induced cell toxicity, ROS generation, and lipid peroxidation and restored GSH depletion, which involved an increase of antioxidant genes through Nrf2 activation, providing evidence that CM-SD can protect cells by reducing oxidative stress. Besides, CM-SD significantly inhibited LPS-mediated IL-6 productions. Therefore, CM-SD could be a useful candidate against oxidative stress and inflammation-related muscle disorders.

## Figures and Tables

**Table 1:** Oligonucleotide sequences used in the present study.

Species	Gene Symbol *	Forward Sequence	Backward Sequence	Product Size (bp)
Mouse	HO-1	5′-GGGAATTTATGCCATGTAAA-3′	5′-AGAACAGCTGCTTTTACAGG-3′	294
	GCLC	5′-GGGTGATCCTCTCATACAAA-3′	5′-GTGTCTATGCTCATCAGGGT-3′	217
	NQO-1	5′-AGGCTGGTTTGAGAGAGT-3′	5′-TCTGCATGCTTTCATCTG-3′	269
	GADPH	5′-AACGACCCCTTCATTGAC-3′	5′-TCCACGACATACTCAGCAC-3′	191

* HO-1, heme oxygenase-1; GCLC, glutamate cysteine ligase catalytic subunit; NQO-1, NAD(P)H-dependent quinone oxidoreductase-1; GAPDH, glyceraldehyde-3-phosphate dehydrogenase.

## Data Availability

Not applicable.
